# Predicting Bitcoin (BTC) Price in the Context of Economic Theories: A Machine Learning Approach

**DOI:** 10.3390/e24101487

**Published:** 2022-10-18

**Authors:** Sahar Erfanian, Yewang Zhou, Amar Razzaq, Azhar Abbas, Asif Ali Safeer, Teng Li

**Affiliations:** 1 Business School, Huanggang Normal University, No. 146 Xinggang 2nd Road, City Development Zone, Huanggang 438000, China; 2 Institute of Agricultural and Resource Economics, University of Agriculture, Faisalabad 38000, Pakistan; 3 Faculty of Behavioral and Social Sciences, University of Groningen, 9712 TG Groningen, The Netherlands; 4 College of Economics and Management, Huazhong Agricultural University, No. 1 Shizishan Street, Hongshan District, Wuhan 430070, China

**Keywords:** AI, business development, information processing, volatility, precision, financial development

## Abstract

Bitcoin (BTC)—the first cryptocurrency—is a decentralized network used to make private, anonymous, peer-to-peer transactions worldwide, yet there are numerous issues in its pricing due to its arbitrary nature, thus limiting its use due to skepticism among businesses and households. However, there is a vast scope of machine learning approaches to predict future prices precisely. One of the major problems with previous research on BTC price predictions is that they are primarily empirical research lacking sufficient analytical support to back up the claims. Therefore, this study aims to solve the BTC price prediction problem in the context of both macroeconomic and microeconomic theories by applying new machine learning methods. Previous work, however, shows mixed evidence of the superiority of machine learning over statistical analysis and vice versa, so more research is needed. This paper applies comparative approaches, including ordinary least squares (OLS), Ensemble learning, support vector regression (SVR), and multilayer perceptron (MLP), to investigate whether the macroeconomic, microeconomic, technical, and blockchain indicators based on economic theories predict the BTC price or not. The findings point out that some technical indicators are significant short-run BTC price predictors, thus confirming the validity of technical analysis. Moreover, macroeconomic and blockchain indicators are found to be significant long-term predictors, implying that supply, demand, and cost-based pricing theories are the underlying theories of BTC price prediction. Likewise, SVR is found to be superior to other machine learning and traditional models. This research’s innovation is looking at BTC price prediction through theoretical aspects. The overall findings show that SVR is superior to other machine learning models and traditional models. This paper has several contributions. It can contribute to international finance to be used as a reference for setting asset pricing and improved investment decision-making. It also contributes to the economics of BTC price prediction by introducing its theoretical background. Moreover, as the authors still doubt whether machine learning can beat the traditional methods in BTC price prediction, this research contributes to machine learning configuration and helping developers use it as a benchmark.

## 1. Introduction

Cryptocurrency is a private system that enables trades between individuals without a central and intermediate agency. In early 2009, Bitcoin (BTC) was valued for the first time at US$0.08. The currency fluctuated for more than four years until the price touched $1110 in 2013. Due to high volatility and massive fluctuations in prices in cryptocurrencies, accurate price predictions are a complex and challenging task. That is mainly because the costs of cryptocurrency move unpredictably and chaotically. Machine learning techniques may help bring in some methodology that will lead to better solutions to the problem. In the last several years, there has been an increasing interest in using machine learning techniques in different areas of science [1,2], particularly cryptocurrency price forecasting [3]. For instance, Dutta et al. [4] used macroeconomic indicators, including interest rates, S&P500 market returns, US bond yields, and gold price level as predictive variables for daily BTC prices. The results show that macroeconomic indicators have short-term predictability power. Wang and Vergne [5] investigated macroeconomic indicators, namely supply growth, defined as BTCs in circulation, to see their effect on BTC return. They found that an increase in supply is positively associated with weekly returns. Conrad et al. [6] found that S&P500 volatility has a significantly positive effect on long-term BTC volatility.

Jang and Lee [7] investigated the effect of blockchain information, including average block size, miner revenue, mining difficulty, and hash rate, on BTC prices. Their results proved that the recent volatility in BTC prices stems from the blockchain information indicators. Wang and Vergne [5] investigated blockchain information indicators, including several unique collaborators contributing code to the project, the number of proposals merged in the core codebase, the number of issues raised by the community about the code, and fixed the developer’s number of forks on BTC returns. They found a positive and significant relationship between blockchain information variables and weekly returns. Therefore, the first research question arises: (1) What are the significant variables as short-term or long-term BTC price predictors? In addition, much previous research on BTC price predictions with machine learning is conducted either using machine learning techniques or conventional statistical analysis without enough theoretical and analytical support. This study investigates whether the macroeconomic, microeconomic, and blockchain information indicators based on economic theories predict the BTC price. According to these considerations, the second research question is: (2) What are the underlying economic theories of BTC price predictors?

There is not enough available literature on BTC price prediction on Google Scholar compared to stocks: around 400 papers about BTC price prediction problems with machine learning algorithms. There are almost 5500 papers about stock price prediction with machine learning algorithms. Also, according to the existing literature, some research on the BTC price prediction problem shows that machine learning outperforms conventional statistical analysis. At the same time, some still believe that traditional models can predict the BTC price better. For instance, Chen et al. [8] applied machine learning techniques models, including random forest, XGBoost, quadratic discriminant analysis, SVM, and LSTM, and statistical methods, including logistic regression and linear discriminant analysis, to predict high-frequency BTC price. They found that Statistical methods achieve an accuracy of 66%, outperforming more complicated machine learning algorithms for daily BTC price prediction. However, machine learning for BTC’s 5-min interval price prediction is superior to statistical methods, with accuracy reaching 67.2%. Pang et al. [9] compared neural network models, sentiment data models, and conventional technical indicators and decision trees to predict BTC prices. The analysis found that the robust neural network models offer better accuracy in predicting BTC prices. Therefore, more research should show whether machine learning algorithms are superior to statistical analysis. Hence, the third research questions are: (3) Are machine learning algorithms superior to traditional methods for BTC price prediction? What machine learning model performs better? What are the best feature selection techniques?

The research innovation herein is looking at BTC price prediction through theoretical aspects. The overall findings show that SVR is superior to other machine learning models and traditional models. This paper has several contributions. It can contribute to international finance to be used as a reference for setting asset pricing and improved investment decision-making. It will be helpful for central bankers, traders, investors, and portfolio managers. Also, it contributes to the economics of BTC price prediction by introducing its theoretical background. Moreover, as the authors still doubt whether machine learning can beat the traditional methods in BTC price prediction, this research contributes to machine learning configuration and helping developers to use it as a benchmark. The rest of the paper is as follows. In the literature review section, there is an overview of existing work and differences from the current work. After that, the methodologies used in this research are briefly explained. Subsequently, the results and discussion are presented. In the end, the paper is concluded.

## 2. Literature Review

The interaction between demand and supply, which determines the price, is critical in economic theory. The theory contrasts the supply side, i.e., the number of coins available in the market, with the demand side, i.e., investors willing to buy. It is the investors or the consumers who are considered the key player. It is assumed that trading in BTC is a reseller market. Reselling to generate profit is the most important in the market. The investors who buy the asset, keep it for a while, and then sell it at a later date are the ones who represent the demand side of this market. BTCs are known for their decentralization as the nodes in the markets are anonymous. Miners are rewarded with BTCs instead of their service for making available the computing power. The miners manage the supply side of BTC, and hence they can be terms as the suppliers as per the whitepaper and the blueprint for BTC, the total supply of BTC will be restricted to 21 million. It is ensured that the mining is gradual and not with large influxes.

In addition, Antoniou et al. [10] describe technical analysis as “part of how traders learn about fundamentals.” The technical analysis predicts future market behavior by studying past market data, such as volume and price. It is based on the premise that historical data can assist in giving future directions. Similarly, Wang and Vergne [5] found a positive correlation between the volume of BTC trading and returns generated. The stated study results concur, proving that technical analysis affects BTC prices.

### 2.1. Underlying Theory of the Macroeconomic Indicators: Demand and Supply Theory

The quantity theory of money is a concept in monetary economics that holds that money’s supply and demand determine the price level. Using this paradigm, Buchholz et al. [11] highlighted how the forces of supply and demand are the main factors influencing the price of Bitcoin. Additionally, utilizing the Keynesian theory of speculative demand for money framework, NaiFovino, et al. [12] and Ciaian et al. [13] highlighted the association between macrofinancial indicators and Bitcoin prices. According to the hypothesis, people who trade in currencies do so to avoid suffering a capital loss on their investments in bonds and other financial assets. A rise in interest rates lowers the value of economic assets, resulting in a loss on the investment of financial assets [14].

Kristoufek [15] extended the research to study the impact of some macroeconomic indicators on the BTC price prediction. He found that Bitcoin appreciates in the long run if it is used more for trade, i.e., non-exchange transactions.

### 2.2. Underlying Theory of the Microeconomic Indicators: Microstructure Theory

The theoretical frameworks of the microstructure approach developed by Lyons [16] imply that the market information structure is asymmetric, which means not all market participants know about the market information. Some agents have their private information, not necessarily about fundamentals. Lyons found that order flow is the most critical determinant of exchange rate determination in the short run. According to Lyons [16], order flow can be measured as the number of buyer-initiated orders minus the number of seller-initiated orders in the market. In microeconomics, supply and demand is an economic market price determination model [17,18]. Theory and empirical evidence suggest that, for an asset with a given cash flow, the higher its market liquidity, the lower its expected return (e.g., [19,20]). Market liquidity affects asset prices and expected returns. In the Bitcoin market, the bid–ask spread factor as a proxy for market liquidity. As more and more buy and sell orders are placed, overall supply and demand become more and more apparent. Some empirical studies also showed the short-term predictability of the Bitcoin microstructure. For example, Dyhrberg et al. [21] investigated the liquidity and transaction costs of Bitcoin markets as a microstructure analysis of Bitcoin. Scaillet et al. [22] showed the bid–ask spread has significant predicting power over jumps in Bitcoin price. In another study, Guo et al. [23] made a short-term prediction of BTC price fluctuations (measured with volatility) using buy and sell orders.

The private information in the BTC market is different from the stock market. In stock market trading, private information is referred to an improved understanding of a firm or company’s prospects and provides a better evaluation of a potential cash flow. When a particular group of traders is made accessible to private information, it helps to create a clear-cut distinction between a BTC market and a stock market. However, it is essential to note that, like the stock market, BTC entertains an uninformed group of traders who enter the market for liquidity only. The questions here follow: What if there remains no future cash flow available for discounting or there remains no asset for valuation? In such a scenario, what exactly would private information provide?

It is indicated that the valuation of BTC is strongly dependent on the level of confidence of its traders. Hence, private information announces great estimation and prediction of the value that a BTC can potentially gain. These types of evaluations are dependent on the consumption of BTC and their usages. Private information like this adds to the prices of BTC and stimulates its demand. Since BTC has a fixed supply, private information helps increase the demand, increasing the prices in the global market. Data provided by the order book covers all the causes of demand and supply conditions of an asset in the form of bids and asks, which are implemented as trades ultimately. The data here provide an insight into the market’s microstructure and an internal overview, which might not be easy to comprehend otherwise. Bid and ask price are two essential components of private information. The bid price refers to the highest price that a potential buyer of BTC is willing to pay. It is also referred to as the buying price for the exchange. When demand for BTC is high, the bid price increases, which means trading volume affects the bid price.

Ask price is the lowest price a seller wants to accept BTC. If the demand falls, there is a fall in the asking price as well. Ask prices are generally higher in comparison to bid prices. Therefore, the difference between these two prices, called the spread, is precisely the profit extracted in these exchanges. BTC prices are highly volatile, which causes extreme fluctuations along with the spreads, which is why sellers enter this market after a great deal of negotiation with the investors and traders to initiate a bidding war. Once that happens, this buying pressure will force an increase price.

### 2.3. Underlying Theory of Blockchain Information Indicators: Cost-Based Pricing Theory

According to Noble and Gruca [24], the cost price of any service or product can be computed based on a predefined profit margin percent calculated over the total cost. The primary focus of the cost-based pricing theory focuses on the variable cost and fixed cost components classified as part of the internal cost. This pricing theory is crucial to BTCs miners as it helps them compute from which cost price is the mining activity more profitable. Blockchain information is one of the critical considerations of BTC’s cost price, as per Wang and Vergne [5]. The mining hardware efficiency can be improved significantly using the right technology resulting in a reduced cost of mining the BTC and a lower price. The lower cost and lower price will lead to increased demand, resulting in ultimately improved return on the overall investment in BTC. Extra hashing power can be achieved for the global mining network on blockchain information which contradicts the lower cost of mining as the difficulty level increases leading to higher mining costs and higher prices for BTCs, resulting in reduced demand and lesser returns.

By developing a cost-of-production model for valuing Bitcoin, Hayes [25] showed that the three factors of computational power, rate of coin production, and mining difficulty used might account for more than 84% of relative value formation. Increasing the difficulty will result in fewer units produced for a given amount of hash power, increasing the relative cost of production. Similar to this, reducing the block reward will result in fewer units. The marginal cost of production is reduced with improved mining hardware energy efficiency, drop-in electricity charges globally, or reduced mining difficulty. With improvement in technical processes, the efficiency of the mining process also improves, which leads to a reduction in the cost of production, which in turn puts downward pressure on prices. In another study, Hayes [26] back-tests a marginal cost of production model applied to value Bitcoin. The author applied vector autoregression (VAR) and traditional regression models on the historical data from 29 June 2013, through 27 April 2018, when the mining difficulty changes, i.e., every two weeks. Results demonstrate that the marginal cost of production is important in explaining Bitcoin pricing in the long run (considering every two weeks a long run prediction).

The block size limits the number of transactions verified with each block, resulting in more computation power for verifying larger blocks. This increased need for more computational power will increase the cryptocurrency price in line with what has been discussed. By definition, hash rate means the quantum of processing and computing power that the mining process contributes to the network. The value of hash rate is referred to provide the value of the network power. Thus computed, this value is used to correct the mining difficulty, i.e., to increase or decrease it and thereby correspondingly increase or decrease the BTC price.

The average block time of the network is evaluated after n number of blocks, and if it is higher than the expected block time, then the level of difficulty of the proof of work algorithm is declined. On the contrary, if the average block time is less than expected, the difficulty level will increase, which is in line with the concept of economics called the law of diminishing marginal utility. The speed with which the things are made available, then the value decreases over time. In terms of BTC terminology, the faster the rate of unit formation, the lower the price of the coin goes.

Difficulty is changed based on the time it took to discover 2016 previous blocks. If a block is found every 10 min (finding 2016 blocks will take exactly 2 weeks). The more (or less) time was spent on finding the previous 2016 blocks the more will difficulty be lowered (raised). Because mining is still lucrative despite the difficulties adjusting higher and the margins becoming somewhat slimmer, more miners are encouraged to join. more miners joining the effort means that the network is growing, which is good for Bitcoin’s price in the long run. This cycle keeps going until a sizable part of the miners can no longer keep up. Some are compelled to sell a growing proportion of the newly created Bitcoins, which finally depletes their treasuries. This causes an increased supply of Bitcoins for sale on the market. They eventually give up and cease mining. The difficulty is then adjusted downward when the hash rate declines.

### 2.4. Application of Machine Learning in Real-World Problem Solving

Artificial intelligence (AI) is a relatively new trend in science that wants to bring about fundamental changes in people’s lives. AI is a little challenging to define, but it can be said that it combines different sciences to make machines more intelligent. One of the most popular subfields of artificial intelligence is machine learning, which is hotly debated. Everyone feels the impact of the learning machine every day in daily life. Simply machine Learning is a science that teaches machines how to learn new things from themselves. Machine learning is one of the modern human inventions that has contributed to the progress of various industries and businesses and has also been very influential in the individual lives of human beings [27]. Machine learning is a subset of artificial intelligence that focuses on learning from the database to build intelligent computer systems. At present, machine learning has been used in various fields and industries. For example, machine learning has been used to diagnose and treat diseases [28], image processing [29], classification [30], and more. Support vector regression can be used in many areas, such as dynamic response prediction of magnetorheological elastomer base isolator [31], thermal spring back of hot press forming [32], text classification [33], etc.

### 2.5. Related Work and Research Gap

Thus far, empirical studies do not demonstrate a clear advantage for the emerging techniques of using machine learning algorithms to predict the BTC price. Research in this area is insufficient [34,35]. Therefore, this study will help to show the significance of machine learning methods in BTC price prediction problems. Also, some research shows machine learning outperforms statistical analysis, and some still believe in the superiority of conventional statistical analysis. Table 1 presents some related work on the BTC price prediction problem. The current research differs from previous studies in terms of completeness and comprehensiveness, and the comparative analysis in the current study has not been conducted before. In addition, a variety of indicators, including macroeconomic indicators, microstructure indicators, blockchain information, and technical indicators, have been used to analyze the significant variables as BTC price predictors.

In the existing literature, there is no comprehensive work in which almost all categories of indicators are investigated. Most of the works regarding BTC price prediction are empirical analyses. However, the current study first looks at the BTC price prediction problem from the perspective of economic theories, including demand and supply theory, microstructure theory, and Cost-based pricing theory. It then identifies the associated variables affecting the BTC price. After that, we empirically prove the predictability power of the attributes through emerging machine learning models and traditional methods.

## 3. Materials and Methods

This research applies a traditional OLS method [45] and some machine learning methods for the BTC price prediction problem, including Ensemble learning, SVR, and MLP multilayer perceptron, which are briefly explained.

### 3.1. Multilayer Perceptron

**Figure 1:**
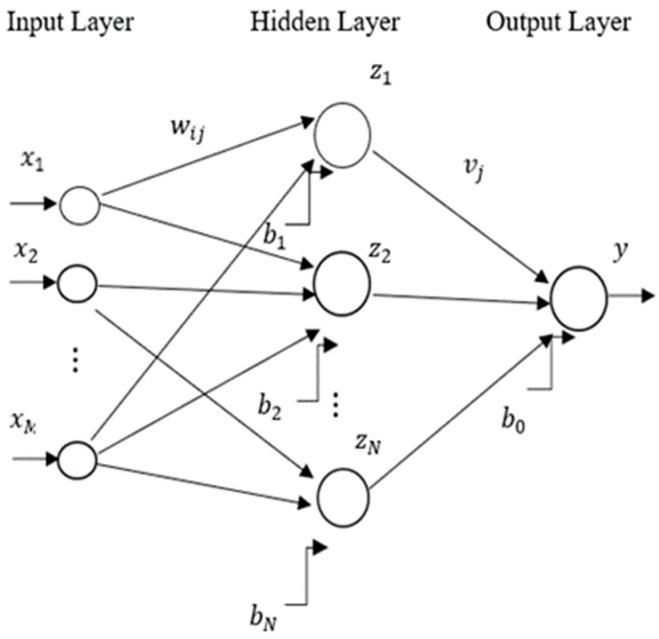
The structure of the three-layer perceptron.

Rosenblatt [46] introduced a multilayer perceptron (MLP) concept with a single perceptron in 1958, consisting of the input layer, middle layers, and output layer. The input layer is a connection between outer space with the network. The middle layers are called hidden layers. Because there is no connection with the outside world, its values are not observed in the training set. The number of neurons in the input layer corresponds to the number of input parameters. Neurons in the hidden layer can be determined by the “trial and error” method. The output layer includes neurons according to our desired output, e.g., the forecasted value in the forecasting problems. A set of weights connects the neurons (see Figure 1).

The output value y of a three-layer perceptron can be formulated as:

(1) $$y=\varphi_{2}(\overset{N}{\underset{j=1}{\sum }}v_{j}z_{j}+b_{0})$$

where N is the number of neurons in the hidden layer, $v_{j}$ is the weight of the second layer, $z_{j}$ is the output of neuron *j*, $b_{0}$ is the bias of the output neuron and $\varphi_{2}$ is the activation function of the output neuron. Several activation functions have been used in MLP models, such as scaled conjugate gradient (SCG), Levenberg–Marquardt (LM), gradient descent with adaptive learning rate (GDA), gradient descent with momentum (GDM), and others. The output value of neuron *j* in the hidden layer is given by:

(2) $$z_{j}=\varphi_{1}\left(\overset{M}{\underset{i=1}{\sum }}w_{ij}x_{i}+b_{j}\right)\;j=1,\ldots ,\;N$$

where M is the number of inputs, $w_{ij}$ are the weights of the first layer, $x_{i}$ are inputs and $b_{j}$ is the bias of neuron *j*, and $\varphi_{1}$ is the activation function of hidden layers. The reason behind choosing MLP is that they are fast to train and can afford hidden layer size 256 instead of 32–64. Also, colossal variance gives a strong ensemble with a single model type.

### 3.2. Support Vector Regression

**Figure 2:**
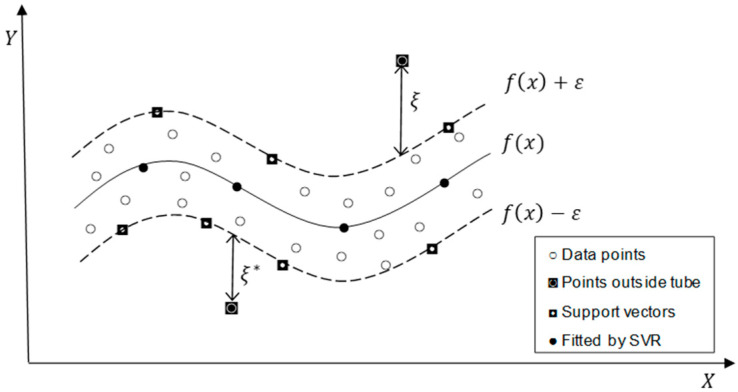
A schematic diagram of the nonlinear regression by SVR based on the ε-insensitive loss function in the feature space.

Support vector regression (SVR) is an emerging nonlinear regression method based on statistical learning theory having a more stable solution than traditional neural network models. Adopting the structural risk minimization principle in SVM reduces overfitting and local minima issues. In SVR, the nonlinear regression problem is transformed into a linear regression problem by mapping the input data into a high dimensional feature space by applying kernel functions [47]. Consider a set of data ${\left(x_{i},y_{i}\right)}_{i=1}^{m}\subset \;{\mathbb{R}}^{m}\times \mathbb{R}$ where $x_{i}$ is a vector of inputs, $y_{i}$ represents the scalar output. In the nonlinear regression case, the linear estimation function can be formulated as $f\left(x\right)=\langle w,\phi \left(x\right)\rangle +b$ where, w∈ ${\mathbb{R}}^{m}$ is weight vector, $\phi \left(x\right)$ is the mapping function, 〈⋅,⋅⟩ denotes the dot product in the feature space, and b is a constant. Several cost functions can be used in SVR, including Huber’s Gaussian, ε-insensitive, and Laplacian. The robust ε-insensitive loss function introduced by Vapnik [48] is the most frequently used function, which can be formulated as follows:

(3) $$L_{\varepsilon }\left(f\left(x\right)-y\right)=\left\{\begin{array}{c} \left|f\left(x\right)-y\right|\;if\;\left|f\left(x\right)-y\right|\;\geq \varepsilon \\ 0\;otherwise\; \end{array}\right.$$

where ε is the tube radius around the regression function *f*(*x*), affecting the number of support vectors used to construct the regression function. The cost of errors on the points inside the tube is zero. Figure 2 shows a schematic diagram of the nonlinear regression by SVR.

The SVR performs linear regression in the feature space using the ε-insensitive loss function by minimizing the empirical risk $R_{emp}=\frac{1}{n}\overset{n}{\underset{i=1}{\sum }}L_{\varepsilon }\left(f\left(x\right)-y\right)$ as well as minimizing the regularization term, $\|w{\|}^{2}$ to reduce the model complexity (flatness). The slack variables $\xi_{i}\;\mathrm{and}\;\xi_{i}^{*}$ represents the deviation of training samples out of the ε-insensitive zone. The optimal regression function can be obtained [47]:

(4) $$min\frac{1}{2}{\left\|w\right\|}^{2}+C\overset{k}{\underset{i=1}{\sum }}(\xi_{i}+\xi_{i}^{*})$$

(5) $$s.t.y_{i}-\langle w,\phi \left(x_{i}\right)\rangle -b\leq \varepsilon +\xi_{i}$$

(6) $$\langle w,\phi \left(x_{i}\right)\rangle +b-y_{i}\leq \varepsilon +\xi_{i}^{*}$$

(7) $$\xi_{i},\xi_{i}^{*}\geq 0$$

where C is the regularization constant determining the trade-off between the empirical risk and the regularization term. The above optimization problem can be solved by using Lagrangian multipliers $\alpha_{i}^{*}$ and $\alpha_{i}$ and Karush–Kuhn–Tucker conditions as the following form:

(8) $$max-\varepsilon \overset{n}{\underset{i=1}{\sum }}(\alpha_{i}^{*}+\alpha_{i})+\overset{n}{\underset{i=1}{\sum }}(\alpha_{i}^{*}-\alpha_{i})y_{i}-\frac{1}{2}\overset{n}{\underset{i,j=1}{\sum }}(\alpha_{i}^{*}-\alpha_{i})(\alpha_{j}^{*}-\alpha_{j})\;K\langle x_{i},x_{j}\rangle$$

(9) $$s.t.\;\overset{n}{\underset{i=1}{\sum }}(\alpha_{i}^{*}-\alpha_{i})=0\;$$

(10) $$\;0\leq \alpha_{i}\leq C,\;i=1,\ldots ,n$$

(11) $$0\leq \alpha_{i}^{*}\leq C,\;i=1,\ldots ,n$$

where $K\langle x_{i},x_{j}\rangle$ is the kernel function which is defined as the inner product of $\phi \left(x_{i}\right)$ and $\phi (x_{j}$) in the feature space. After solving the optimization problem, the optimal form of the regression function can be obtained as [47]:

(12) $$f\left(x\right)=\overset{n}{\underset{i=1}{\sum }}\left(\alpha_{i}-\alpha_{i}^{*}\right)K\;\langle x,x_{i}\rangle +b$$

By setting the parameters C and ε and the kernel parameters, the estimation accuracy can be obtained. The reason for choosing SVR is that it is robust to outliers. The decision model can be easily updated. It has excellent generalization capacity with high prediction accuracy, and its implementation is straightforward.

### 3.3. Ensemble Method

Various experiences show no specific training algorithm in machine learning methods that can be the best and most accurate for all applications. Each algorithm is a particular model based on certain assumptions. Sometimes these assumptions are met, and sometimes they are violated. Therefore, no algorithm alone can succeed in all situations. Ensemble methods have been introduced to solve this problem. The primary motivation for developing the Ensemble method is to reduce the error rate. Forecasting error using the Ensemble approach, a group of techniques is much lower than using a single model. When combining independent and different classifiers, the likelihood of making the right decision is strengthened since each of these classifiers will perform better than a random guess.

**Figure 3:**
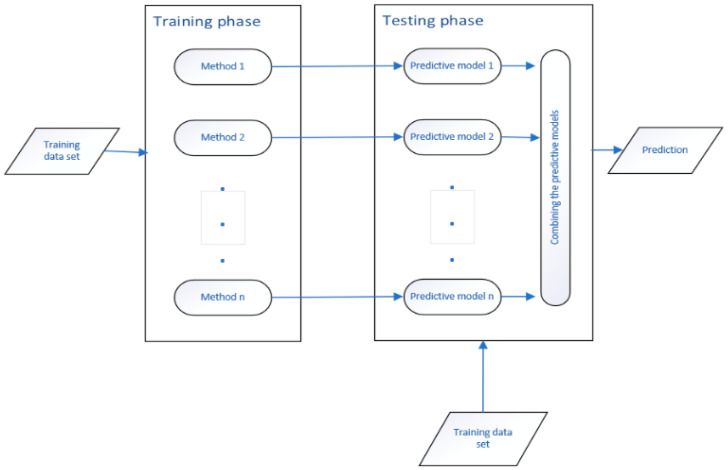
Ensemble method flowchart.

Hansen and Salamon [49] presented deploying multiple models on regression. They proved that someone could show that the overall error E decreases uniformly concerning N with the N independent classifier with a probability of error e < 0.5. Also, the overall performance is significantly reduced if someone uses dependent categories. The methodology consists of two consecutive steps: The training and testing phases. As shown in Figure 3, several predictive models are produced using training samples in the training phase. Predictive models would combine to predict the next step or the testing phase.

Some popular ensemble methods are Boosting, Bagging, and Blending, of which the Bagging approach will be used in this research. There are two main reasons to choose an Ensemble model: performance and robustness. The Ensemble model can make better forecasts and do better than any single model. An Ensemble model reduces the spread or distribution of the estimates and model accuracy.

### 3.4. Feature Selection Methods

**Figure 4:**
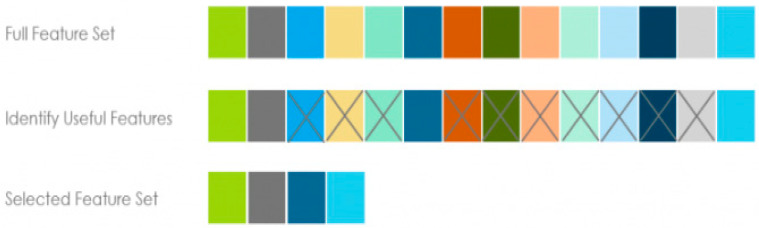
Feature selection in one glance (each color is representing one feature).

Feature selection, variable selection, or attribute selection plays an essential role in classification problems. It reduces the number of attributes by excluding the irrelevant and redundant ones to achieve the lower complexity model (see Figure 4). The more uncomplicated and faster models with fewer variables are desirable in machine learning models. Feature selection is an essential part of the machine learning process, leading to overfitting. Overfitting happens when the model learns details and noises made by too many variables, and then the model will not generalize well when presented with new data.

In this research, some feature selections, such as principal component analysis (PCA), particle swarm optimization (PSO), evolutionary search, genetic search, best-first search, and variance inflation factor (VIF), are used.

### 3.5. Model Evaluation

A model evaluation metric quantifies a predictive model’s performance, typically involving training a model on a dataset, using the model to make predictions on a “test dataset” not used during training, then comparing the predictions to the expected values in the test dataset. Different authors use different metrics to compare their models. Table 2 shows the evaluation metrics used in this study. In all formulas, $y_{t}$ ${\hat{y}}_{t}$ *T* is the target value, output value, and the size of a test dataset in out-of-sample or out-of-fold prediction.

### 3.6. Model Validation

One of the more used statistical analyses, cross-validation, helps assess and validate the machine learning model’s performance. The key intention behind evaluating the model is to see whether or not one can check if the trained model is generalizable. As part of the K-fold cross-validation process, the entire data set is first split into several folds. After that, the model is trained on all folds but one and the test model on the remaining fold. The test is reiterated multiple times until the model tests all the folds. Finally, the average scores obtained in every fold are taken as the final metrics. Predictions are made on the test sets that were not used to train the model during the process. These predictions are called ‘out of fold predictions,’ a type of ‘out of the sample’ forecast. In contrast to the simple train-test, the method discussed prevents overfitting and helps in a more robust model evaluation form.

**Figure 5:**
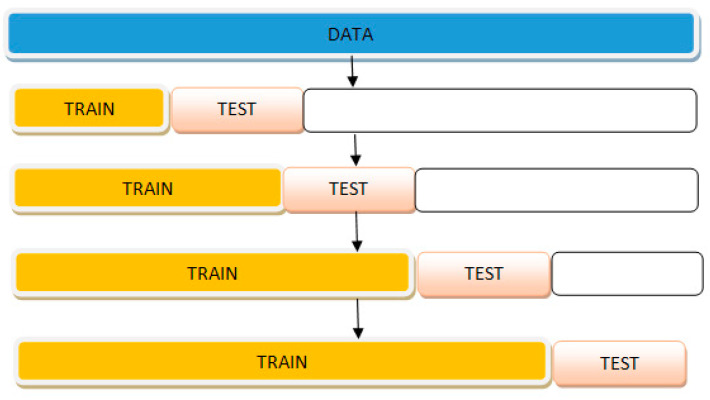
Cross-validation on a rolling basis.

Cross-validation on a rolling basis is a method that is used for cross-validating the time series models. According to Kuhn and Johnson [52], the value of *k* = 10 is expected. The repeated K-fold cross-validation method replicates the entire process multiple times. For instance, if ten-fold cross-validation were repeated five times, it would result in 50 times out-of-fold predictions, estimating the model’s efficacy. The ten times K-fold cross-validation is a prevalent method to Kuhn and Johnson [52]. As depicted in Figure 5, the process starts with a small subset of data for training. Subsequently, the forecast for the later data point finally, the data point is for checking the accuracy. The same forecasted data point is included in the following training data set basis on which the next data points are predicted.

## 4. Results and Discussion

This section consists of three parts. In the first part, a multilinear regression model is built for the BTC price prediction problem on monthly BTC prices from 18 August 2010 to 17 September 2018. Data includes macroeconomic and blockchain information indicators. The second part presents two comparative approaches: feature-based and category-based comparative analysis consisting of OLS, Ensemble methods, SVR, and MLP for the BTC price prediction problem on a daily data set from 11 October 2016 to 12 June 2017. Data is composed of macroeconomic, microeconomic, and technical indicators. All predictions in this part are out-of-fold predictions.

During the k-fold cross-validation process, predictions are made on test sets comprised of data not used to train the model. These predictions are called out-of-fold predictions, a type of out-of-sample predictions. Another analysis similar to the second part is described in the third part on different BTC datasets, including macroeconomic, microeconomic, blockchain information, and technical indicators from 1 January 2018 to 5 June 2018. For validation of results in this research, three metrics, namely RMSE, R^2^, and Pearson *r*, have been used to compare the out-of-sample and out-of-fold predictive models under the *T*-test at the significance level of 0.05. The *k*-fold cross-validation with *k* = 10 (so-called cross-validation on a rolling basis) is used to construct a high-performance model and have robust results. Results are averaged on 100 prediction trials.

### 4.1. The BTC Price Prediction Problem Using OLS

According to the theoretical analysis regarding demand and supply theory, macroeconomic indicators have long-term predictability power on BTC prices. For the empirical analysis, a multilinear regression model is built for the BTC price prediction problem (model 1 in the Appendix A) on monthly BTC prices from 18 August 2010 to 17 September 2018, including macroeconomic and blockchain information indicators.

#### 4.1.1. Data Description

Monthly BTCUSD transactions occurring on the significant BTC exchanges, available at blockchain.com from 18 August 2010 and ending on 17 September 2018, including 24 variables, have been examined. Dependent variables can be categorized into Macroeconomic indicators and Blockchain information indicators obtained via provided API at blockchain.com (see Table 3). Some descriptive statistics, including minimum, maximum, mean, and standard deviation, have been calculated and shown in Table A1 in the Appendix A.1 to describe or summarize the data.

#### 4.1.2. Feature Selection

First, data cleaning, including estimating outliers (extreme values) and missing values, has been applied to the raw data to build a better data set. After that, VIF is applied to the data set to deal with multicollinearity. Table A2 in the Appendix A.1 shows variables, namely, market capitalization, transactions per block, Hash Rate, mining difficulty, cost per transaction, total transactions per day, Nasdaq Composite, Dow Jones Industrial Average, and S&P 500, which have a VIF greater than 10. Instead of dropping variables, the entire sample period has been tested in nine models with different combinations of variables.

#### 4.1.3. OLS Regression for BTC Price Prediction

Table A3 in the Appendix A.1 shows the results of nine regression models built to avoid multicollinearity. The variables in quotes are the variables with a high correlation. They are added to the rest of the variables to build a new regression model. The response variable in each model is the BTC price. The value in parentheses represents the results of the *t*-test for the null hypothesis-rejecting variables, based on a *p*-value of 0.05. The *R*^2^ from regression models is relatively high, suggesting that, for example, approximately 73% of the variation in BTC prices in model “9” is determined by the variables in the model. Due to the t-statistics and p-value, all models are statistically significant. By looking at the coefficients, which are not tiny, it is evident that all variables are economically significant for the models.

The regression analysis showed that the significant macroeconomic indicators in all models for monthly BTC price are market capitalization, Nasdaq Composite, Dow Jones Industrial Average, and S&P500. Therefore, macroeconomic indicators have long-term predictive power on BTC prices as expected a priori and the t-statistic indicates the significance of the results. Also, blockchain information indicators, including the block size, cost per transaction, mining difficulty, hash rate, transaction fees, and estimated transaction value, verify that the supply and demand theory is the underlying theory of predictors. Therefore, blockchain information indicators have a long-term predictive power on BTC prices as expected a priori. The t-statistic indicates that it is highly statistically significant that blockchain information indicators influence the price confirming that the cost-based pricing theory is underlying the predictors. Empirical results answer the first and second research questions. (1) What are the significant variables as short-term or long-term BTC price predictors? (2) What are the underlying economic theories of BTC price predictors?

### 4.2. Proposed Comparative Analysis for Dataset 1

According to the theoretical analysis regarding demand and supply theory, macroeconomic indicators do not have short-term predictability power on BTC prices. For the empirical analysis, a comparative machine learning model, including OLS, Ensemble methods, SVR, and MLP for the BTC price prediction problem on data sets from 11 October 2016, to 12 June 2017, including macroeconomic, microeconomic, and technical indicators. Feature selections, namely Best First Search, PSO Search, and Evolutionary Search, are applied to the data. The price prediction model is described in the Appendix A (model 2).

#### 4.2.1. Data Description

Daily BTC/USD transactions occurring on the Bitfinex exchange, obtained via provided API at bitfinex.com (accessed on 2 October 2019) from 11 October 2016, to 12 June 2017, including 22 independent variables, have been examined. Dependent variables can be categorized into three groups; Macroeconomic indicators, obtained at fred.stlouisfed.org, and microeconomic and technical indicators extracted from bitfinex.com. Table 4 shows the specification for each group. Some descriptive statistics, including minimum, maximum, mean, and standard deviation, have been calculated and shown in Table A4 in the Appendix A.2 to describe or summarize the data.

#### 4.2.2. Feature-Based Comparative Analysis

This section applies the comparative analysis to different datasets containing the indicators chosen by different feature selection techniques, including VIF, genetic search, evolutionary search, and best-first search. Table A5 in the Appendix A.2 shows the different features chosen by various methods. The comparison is conducted under the *T*-test at the significance level of 0.05 by WEKA software (version 3.9.4, developed at the University of Waikato, New Zealand). To evaluate the predictive machine learning models’ performance and have robust results, the 10-fold cross-validation on a rolling basis evaluation technique is used, and each model is repeated ten times. Therefore, the average results of 100 prediction trials, including the forecasting ability of models, namely RMSE and Pearson’s *r*, are shown in Table 5 and Table 6. The standard deviation is shown in parenthesis.

According to Table 5 and Table 6, the SVR performs better on the attributes made by PCA. Thus, one can use a combination of SVR and PCA to boost the model. No feature selection can improve the models. The VIF method is the worst feature selection method among the mentioned feature selection methods due to the poor prediction results. Different models are compared to identify the best model for each data set, except for VIF data (due to the not promising forecasting results). Table 7 summarizes the model’s comparisons, showing that the SVR model has the best accuracy and the MLP has the worst accuracy.

#### 4.2.3. Category-Based Comparative Analysis

This section applies the comparative analysis to different datasets containing different categories such as macroeconomic, microeconomic, and technical indicators. Comparison is conducted under the *T*-test at the significance level of 0.05 by WEKA software. To evaluate the predictive machine learning models’ performance and have robust results, the 10-fold cross-validation on a rolling basis evaluation technique is used, and each model is repeated ten times. Therefore, the average results of 100 prediction trials, including the forecasting ability of models, namely RMSE and Pearson’s *r*, are shown in Table 8 and Table 9. The standard deviation is represented in parenthesis.

According to Table 8 and Table 9, technical indicators impact prediction results in OLS and SVR models. The Ensemble methods and MLP models have the best accuracy on the data, including all variables. Prediction using technical indicators or using all indicators has nearly the same accuracy. In addition, all models applied on the macroeconomic and microeconomic indicators have bad accuracy with a very low Pearson’s *r* and high RMSE. Therefore, it is not recommended to be used. The order of indicators according to their impact on prediction is shown in Table 10. Models applied to all attributes, and technical indicators are compared in Table 11. In both cases, the SVR model outperforms other models. Also, MLP is considered the worst model.

The category-based comparative analysis showed that macroeconomic indicators (trade-weighted US dollar index, gold fixing price, DJIA index, Brent crude oil price, and WTI) are not significant predictors for short-term BTC price. Microeconomic indicators are also not significant except for the MLP model. In addition, technical indicators, namely volume, MTM, CCI, and SMA, predict the price with nearly the same accuracy as the prediction model using all indicators. Therefore, the recommendation is to use technical analysis to predict the short-term BTC price. These empirical results answer the first and second research questions. (1) What are the significant variables as short-term or long-term BTC price predictors? (2) What are the underlying economic theories of BTC price predictors? To answer the third research question (What machine learning model performs better? What are the best feature selection techniques?), empirical results showed that the SVR model in feature-based and category-based comparative analyses outperform other models. Also, in terms of data preparation, no feature selection improved the model, and VIF turned out to be the worst feature selection.

### 4.3. Proposed Comparative Analysis for Dataset 2

According to the theoretical analysis regarding demand and supply theory and cost-based pricing theory, macroeconomic and blockchain information indicators do not have short-term predictability power on BTC prices. For the empirical analysis, a comparative machine learning model, including OLS, Ensemble methods, SVR, and MLP for the BTC price prediction problem on datasets from 1 January 2018 to 5 June 2018, including macroeconomic, microeconomic, technical indicators, and blockchain information indicators. Feature selections, namely best first search, PSO search, and evolutionary search, are applied to the data. The price prediction model is described in the Appendix A (model 3).

#### 4.3.1. Data Description

Daily BTCUSD transactions occurring on the Bitfinex exchange obtained via provided API at bitfinex.com from 1 January 2018, to 5 June 2018, including 17 independent variables, have been examined. Dependent variables can be categorized into macroeconomic variables, extracted from macrotrends.net (accessed on 2 October 2019), microeconomic, technical indicators, and Blockchain information indicators obtained from data.BTCity.org. Table 12 shows the specification for each group. Some descriptive statistics, including minimum, maximum, mean, and standard deviation, have been calculated and shown in Table A6 in the Appendix A.3 to describe or summarize the data.

#### 4.3.2. Feature-Based Comparative Analysis

This section applies the comparative analysis to different datasets containing the indicators chosen by different feature selection techniques, including best-first search, evolutionary search, PSO search, and PCA dimension reduction methods. Table A7 in the Appendix A.3 presents the different features chosen by other methods. For the analysis, machine learning models, including OLS, Ensemble methods (bagging), SVR (with a polynomial kernel), and MLP (with one hidden layer and nine neurons), have been applied to different datasets, which include the indicators selected by other feature selections. The aim is to specify the best feature selection method and determine the best machine learning method. To evaluate the predictive machine learning models’ performance and have robust results, the 10-fold cross-validation on a rolling basis evaluation technique is used, and each model is repeated ten times. Therefore, the average results of 100 prediction trials, including the forecasting ability of models, namely RMSE and Pearson’s *r*, are shown in Table 13 and Table 14.

According to Table 13 and Table 14, all models applied to all indicators have the best accuracy than those applied to the other datasets. Therefore, it can be concluded that no feature selection improves the model’s accuracy. Compared to those applied to the different datasets, all models applied to data reduced by PCA have the lowest accuracy. Therefore, it can be concluded that the PCA reduction method is not a promising feature selection method for this research data. Different models are compared together for each data set to identify the best model. Table 15 summarizes the model’s comparisons, showing that the SVR model has the best accuracy for all datasets, and the MLP has the least accuracy.

#### 4.3.3. Category-Based Comparative Analysis

OLS, Ensemble methods, SVR, and MLP are applied to economic and technical indicators. The aim is to see which indicators can be selected as better predictive indicators. Also, different models are compared on the same data to find a more accurate model. To evaluate the predictive machine learning models’ performance and have robust results, the 10-fold cross-validation on a rolling basis evaluation technique is used, and each model is repeated ten times. Therefore, the average results of 100 prediction trials, including the forecasting ability of models, namely RMSE and Pearson’s *r*, are shown in Table 16 and Table 17.

According to Table 16 and Table 17, all models applied to all indicators have the best accuracy. Therefore, it is recommended that the combination of technical, microeconomics, macroeconomic, and Blockchain information indicators work better for price prediction than each indicator category alone. Moreover, technical indicators are also considered good predictors. However, prediction slightly improves by combining with other variables. Blockchain information and macroeconomic indicators are considered bad predictive indicators due to the very low Pearson’s *r* and high RMSE. The order of indicators according to their impact on prediction is shown in Table 18. Models applied on all indicators and technical indicators are compared in Table 19. In both cases, the SVR model outperforms other models. Also, MLP is considered the worst model.

The results of the category-based comparative analysis showed that macroeconomic indicators (trade-weighted US dollar index, gold-fixing price, DJIA index, Brent crude oil price, and WTI) are not significant predictors. Also, the Blockchain information indicators, including hash rate, mining difficulty, number of transactions per block, and block time, are not significant predictors for short-term BTC price. Also, microeconomic indicators, including trades per minute, bid/ask sum, bid–ask spread, and buy/sell signals, are not significant for the BTC price prediction except for the MLP model. Since the technical indicators have nearly the same results as all indicators, the recommendation is to use the technical analysis to predict the short-term BTC price. These empirical results answer the first and second research questions. (1) what are the significant variables as short-term or long-term BTC price predictors? (2) What are the underlying economic theories of BTC price predictors? To answer the third research questions (What machine learning model performs better? What are the best feature selection techniques?), empirical results showed that the SVR model in feature-based and category-based comparative analyses outperform the other models. In terms of data preparation, no feature selection improved the model, and PCA dimension reduction turned out to be the worst feature selection.

## 5. Conclusions

Today, international finance is a multi-trillion-dollar sector that needs a secure and stable mechanism that cryptocurrencies are currently inching. Cryptocurrencies were developed under Blockchain technology. In contrast with the traditional central authority systems wherein the sole control lies under one organization, Blockchain technology has a diversified approach. This paper applied several machine learning models to the BTC price prediction model on different data sets to verify the theoretical analysis and answer the research questions. A multilinear regression model to monthly BTC prices showed that macroeconomic and Blockchain information indicators are significant long-term predictors. That verifies that supply and demand and cost-based pricing theory are underlying BTC price predictors. These empirical results answer the first and second research questions. (1) What are the significant variables as short-term or long-term BTC price predictors? (2) What are the underlying economic theories of BTC price predictors? In addition, the empirical results showed that SVR is the best machine learning model, and no feature selection technique is proven to be the best, which answers the third research questions (Are machine learning algorithms superior to traditional methods for BTC price prediction? What machine learning model performs better? What are the best feature selection techniques?).

The conclusions are relevant to central bankers, investors, asset managers, etc., who are generally interested in information about which indicators provide reliable, accurate forecasts of BTC price. The study can be used to set asset pricing and improve investment decision-making. Therefore, it provides a significant opportunity to contribute to international finance since the results have significant implications for the future decisions of asset managers. In time series prediction, the correlation between independent variables and dependent variables differs from time to time. Consequently, reestimating prediction models is not unlikely. This study has used many data categories composing macroeconomic, microstructure, Blockchain information, and technical indicators to make a wide-ranging work.

In this study, attributes are selected based on economic theories. Macroeconomic indicators are chosen based on the supply and demand theory. Microstructure theory is the underlying theory of microeconomic indicators. Also, Blockchain information indicators are selected according to the cost-based pricing theory. Previous studies are mostly empirical research in which the focus is on the prediction methods. After describing the price movement from the perspective of economic theories, the empirical results confirmed the theoretical analysis. This study compared methodologies to predict short-term and long-term BTC prices. The conclusion is also helpful for machine learning developers to understand the configuration of machine learning prediction models and use it as benchmarks. According to the literature review, the authors still doubt whether machine learning can beat the traditional methods for BTC price prediction. Therefore, this study is evidence of the superiority of machine learning.

This research has some suggestions for future work, which are as follows. In this research, only a few critical feature selection methods have been applied to data sets. Many other attribute selection techniques, including ranker search, Tabu search, and many more, can be examined to improve the model. Other research can compare trending models, such as recurrent neural networks (RNN) to SVR. According to this research, a correct prediction of BTC prices can be profitable; therefore, it can diversify a portfolio. Further studies can be conducted to examine the portfolio return by adding BTC to a portfolio to determine the right amount of BTC to keep. Future research can predict other cryptocurrencies, including Ethereum and Ripple. In addition, some other indicators, such as “news,” can be investigated in other studies.

## Figures and Tables

**Table 1:** Overview of research published on BTC price prediction.

Reference	Year	Methodologies	Data Categorization	Findings
Chen et al. [8]	2020	Logistic Regression and Linear Discriminant Analysis, Random Forest, XGBoost, Quadratic Discriminant Analysis, SVM, and Long Short-term Memory	Blockchain Information, Macroeconomic Indicators	Statistical methods outperform machine learning for BTC daily price prediction, while, Machine learning for BTC’s 5-min interval price prediction is superior to statistical methods,
Aggarwal et al. [36]	2020	SVM and decomposition (CEEMD)	technical indicators	The proposed method for short-term, midterm, and long term-prediction has a predictability power
Dutta et al. [4]	2020	Gated Recurring Unit, simple neural network (NN), LSTM	Blockchain Information, Macroeconomic Indicators, Technical Indicators	GRU outperforms the NN and LSTM for daily price prediction
Jiang, X. [37]	2019	MLP, LSTM, Gated Recurrent Network	Technical Indicators	
Munim et al. [38]	2019	ARIMA and neural network autoregression (NNAR)	Technical Indicators	ARIMA outperforms NNAR in daily price prediction
Huang et al. [39]	2019	A tree-based predictive mod buy and-hold strategy	Technical Indicators,	A tree-based predictive model for daily return outperform a buy and-hold strategy
Shen et al. [40]	2019	GARCH model, SMA, RNN	Technical Indicators	RNN method outperforms the GARCH model and SMA model for daily return prediction
Mangla et al. [41]	2019	Logistic regression, SVM, RNN, and ARIMA	Technical Indicators	ARIMA is better for next-day prediction, RNN better for weekly
Siami-Namini and Namin [42]	2018	ARIMA, long short-term memory (LSTM)	Technical Indicators	LSTM is superior to ARIMA for daily prediction
Jang and Lee [7]	2017	Bayesian neural networks (BNNs), SVR, and linear regression	Blockchain Information and macroeconomic indicators	BNN outperforms SVR and linear regression
Pichl and Kaizoji [43]	2017	Multilayer Perceptron	Technical Indicators	HARRVJ neural network captures well the dynamics of daily Realized Volatility as aggregated on the 5-min grid.
Indera et al. [44]	2017	MLP-based NARX	Technical Indicators	NARX has predictive power for daily price
Current Work	2022	OLS, MLP, ENSEMBLE, and SVR	Technical indicators, macroeconomic indicators, microstructure indicators, and blockchain information indicators	SVR beats the other models Macroeconomics and blockchain information have long term predictivity power There is no feature selection to improve the model

**Table 2:** Common types of evaluation metrics.

Accuracy Metrics	Formula
$R^{2}$ [50]	$R^{2}=1-\overset{T}{\underset{t=1}{\sum }}{\left({\hat{y}}_{t}-y_{t}\right)}^{2}/\overset{T}{\underset{t=1}{\sum }}{\hat{y}}_{t}{}^{2}$ *T* is the size of a test dataset in out of sample prediction
Pearson’s *r*	$r=\overset{T}{\underset{t=1}{\sum }}{\hat{y}}_{t}y_{t}/\sqrt{\overset{T}{\underset{t=1}{\sum }}{\hat{y}}_{t}{}^{2}}\sqrt{y_{t}}$
Root Mean Square Error (RMSE) [51]	$\mathrm{RMSE}=\sqrt{\overset{T}{\underset{t=1}{\sum }}{\left(\hat{y_{t}}-y_{t}\right)}^{2}/T}$

**Table 3:** Data categorization.

Indicator Category	Indicator Name
Macroeconomic indicators	Market capitalization, BTCs in circulation, US federal funds rate, S&P500 stock market index, Nasdaq composite, DJIA stock market index, WTI, gold-fixing price, breakeven inflation rate,
Blockchain information indicators	Hash rate, mining difficulty, number of transactions per block, block size, average block size, median confirmation time, orphan blocks, cost per transaction, transaction fees, estimated transaction value (BTC), estimated transaction value (USD), total output value

**Table 4:** Data categorization.

Indicator Category	Indicator Name
Macro-Economic Indicators	Trade-weighted US Dollar Index, gold-fixing price, DJIA Index, Brent Crude oil price, WTI
Microeconomic Indicators	Trades per minute, bid/ask sum, bid–ask spread, buy/sell signals,
Technical Indicators	volume, MTM, CCI, SMA

**Table 5:** RMSE of different models on different data sets.

Model Indicators	OLS	Ensemble Methods (Bagging)	SVR	MLP
All indicators	8.86 (2.36)	9.04 (1.97)	8.68 (2.48)	9.30 (2.20)
PCA Reduction	8.79 (1.98)	11.45 (2.48)	8.59 (2.09)	11.67 (2.31)
VIF	15.97 (3.03)	13.92 (3.00)	16.01 (3.18)	15.28 (4.57)
Genetic Search	8.77 (2.23)	9.45 (2.05)	8.67 (2.27)	10.11 (2.39)
Evolutionary Search	8.72 (1.98)	9.00 (2.06)	8.68 (2.13)	9.56 (2.39)
Best First	8.80 (2.23)	9.40 (2.07)	8.68 (2.26)	10.08 (2.49)

**Table 6:** Pearson’s *r* of different models on different indicators.

Model Indicators	OLS	Ensemble Methods (Bagging)	SVR	MLP
All Indicators	0.88 (0.08)	0.88 (0.07)	0.89 (0.08)	0.89(0.07)
PCA	0.88 (0.06)	0.88 (0.07)	0.89 (0.07)	0.80(0.09)
VIF	0.56 (0.15)	0.68 (0.17)	0.55 (0.15)	0.72(0.15)
Genetic Search	0.88 (0.07)	0.87 (0.07)	0.88 (0.07)	0.87(0.07)
Evolutionary Search	0.88 (0.07)	0.88 (0.07)	0.89 (0.07)	0.88(0.06)
Best First Search	0.88 (0.07)	0.87 (0.07)	0.88 (0.07)	0.87(0.07)

**Table 7:** Order of the models in terms of the accuracy.

Indicators	Models
All Indicators	SVR, OLS, Ensemble methods, and MLP
PCA	SVR, OLS, Ensemble methods, and MLP
Genetic Search	SVR, OLS, Ensemble methods, and MLP
Evolutionary Search	SVR, OLS, Ensemble methods, and MLP
Best First Search	SVR, OLS, Ensemble methods, and MLP

**Table 8:** RMSE of different models on different indicators.

Model Indicators	OLS	Ensemble Methods (Bagging)	SVR	MLP
All indicators	8.86 (2.36)	9.04 (1.97)	8.68 (2.48)	9.30 (2.20)
Macroeconomic indicators	19.27 (3.55)	18.54 (3.97)	19.25 (3.79)	20.74 (4.42)
Microeconomic indicators	18.42 (3.76)	16.04 (2.83)	18.76 (3.99)	17.35 (4.02)
Technical indicators	8.72 (2.10)	9.05 (2.14)	8.68 (2.17)	9.61 (2.39)

**Table 9:** Pearson’s *r* of different models on different indicators.

Model Indicators	OLS	Ensemble Methods (Bagging)	SVR	MLP
All Indicators	0.88 (0.08)	0.88 (0.07)	0.89 (0.08)	0.89 (0.07)
Macroeconomic Indicators	0.06 (0.19)	0.25 (0.29)	0.09 (0.27)	0.25 (0.22)
Microeconomic Indicators	0.33 (0.19)	0.53 (0.23)	0.27 (0.21)	0.61 (0.20)
Technical Indicators	0.88 (0.07)	0.88 (0.07)	0.88 (0.07)	0.88 (0.07)

**Table 10:** The order of indicators according to their impact on prediction.

Models	The Order of Indicators according to Their Impact on Prediction
OLS	Technical indicators, all indicators, microeconomic indicators, macroeconomic indicators
Ensemble methods	All indicators, technical indicators, microeconomic indicators, macroeconomic indicators
SVR	Technical indicators, all indicators, microeconomic indicators, macroeconomic indicators
MLP	All indicators, technical indicators, microeconomic indicators, macroeconomic indicators

**Table 11:** The order of the models in terms of accuracy.

Indicators	Models
All Indicators	SVR, OLS, Ensemble methods, and MLP
Technical Indicators	SVR, OLS, Ensemble methods, and MLP

**Table 12:** Data categorization.

Indicator Category	Indicator Name
Macroeconomic indicators	S&P500 index, Nasdaq Composite, DJIA index, CAC 40 Index, WTI, gold fixing price
Microeconomic indicators	Bid–ask spread (10BTC), ask sum (10%), bid sum (10%), trades per minute
Technical indicators	Volatility, volume, SMA
Blockchain information indicators	Hash rate, mining difficulty, number of transactions per block, block time

**Table 13:** RMSE of different models on different datasets.

Model Indicators	OLS	Ensemble Methods (Bagging)	SVR	MLP
All Indicators	157.36 (30.24)	160.06 (36.52)	154.49 (31.53)	163.37 (44.62)
Best First	161.36 (34.57)	162.85 (38.69)	158.87 (36.20)	164.16 (40.37)
PCA Reduction	160.48 (34.38)	178.77 (40.04)	160.26 (33.52)	179.77 (45.12)
PSO Search	160.70 (29.31)	162.90 (37.43)	158.06 (34.26)	175.40 (43.50)
Evolutionary Search	161.03 (31.97)	162.43 (34.99)	160.00 (38.76)	169.70 (49.65)

**Table 14:** Pearson’s *r* of different models on different data sets.

Model Indicators	OLS	Ensemble Methods (Bagging)	SVR	MLP
All Indicators	0.77 (0.13)	0.74 (0.14)	0.76 (0.13)	0.77 (0.12)
Best First Search	0.74 (0.16)	0.72 (0.16)	0.74 (0.14)	0.77 (0.16)
PCA Reduction	0.76 (0.13)	0.65 (0.17)	0.74 (0.13)	0.76 (0.12)
PSO Search	0.75 (0.14)	0.72 (0.16)	0.75 (0.13)	0.73 (0.17)
Evolutionary Search	0.74 (0.15)	0.74 (0.13)	0.74 (0.14)	0.77 (0.16)

**Table 15:** Order of the models in terms of accuracy.

Datasets	Models
All Indicators	SVR, OLS, Ensemble methods, and MLP
Best First Search	SVR, OLS, Ensemble methods, and MLP
PCA Reduction	SVR, OLS, Ensemble methods, and MLP
PSO Search	SVR, OLS, Ensemble methods, and MLP
Evolutionary Search	SVR, OLS, Ensemble methods, and MLP

**Table 16:** RMSE of different models on different indicators.

Model Indicators	OLS	Ensemble Learning	SVR	MLP
All indicators	157.36 (30.24)	160.06 (36.52)	154.49 (31.53)	174.37 (44.62)
Blockchain information indicators	242.29 (46.77)	243.07 (48.78)	248.09 (51.72)	281.13 (60.84)
Macroeconomic indicators	251.56 (46.90)	230.01 (43.84)	249.30 (47.05)	262.18 (59.76)
Microeconomic indicators	198.61 (36.65)	193.00 (36.62)	197.99 (37.74)	205.60 (48.95)
Technical indicators	173.07 (41.11)	161.97 (38.69)	172.72 (40.78)	191.32 (52.98)

**Table 17:** Pearson’s *r* of different models on different indicators.

Models Indicators	OLS	Ensemble Learning	SVR	MLP
All indicators	0.75 (0.13)	0.74 (0.14)	0.76 (0.13)	0.77 (0.12)
Blockchain information indicators	0.11 (0.27)	0.10 (0.24)	−0.01 (0.25)	−0.04 (0.26)
Macroeconomic indicators	−0.00 (0.25)	0.23 (0.34)	0.07 (0.32)	0.21 (0.31)
Microeconomic indicators	0.57 (0.23)	0.58 (0.21)	0.57 (0.22)	0.60 (0.23)
Technical indicators	0.68 (0.16)	0.73 (0.14)	0.69 (0.16)	0.69 (0.16)

**Table 18:** The order of indicators according to their impact on prediction.

Models	Order of Indicators according to Their Impact on Prediction
OLS	All indicators, technical indicators, microeconomic indicators, blockchain information indicators, macroeconomic indicators
Ensemble methods	All indicators, technical indicators, microeconomic indicators, macroeconomic indicators, Blockchain information indicators
SVR	All indicators, technical indicators, microeconomic indicators, Blockchain information indicators, macroeconomic indicators
MLP	All indicators, technical indicators, microeconomic indicators, macroeconomic indicators, Blockchain information indicator

**Table 19:** The order of the models in terms of accuracy.

Indicators	Models
All indicators	SVR, OLS, Ensemble methods, and MLP
Technical indicators	SVR, OLS, Ensemble methods, and MLP

## Data Availability

The datasets used and analyzed during this study are available from the corresponding author on reasonable request.

## Appendix A

### Appendix A.1. Model 1. OLS Model Description

The purpose is to find a model that can approximate a target function, which can be written as:

(A1) $$r_{i+1}=\alpha +\beta_{1}X_{i,1}+\beta_{2}X_{i,2}+.+\beta_{N}X_{i,N}+\varepsilon_{i}\;$$

where $r_{i}$ is the BTC price at month i+1. $X_{i,1}\;to\;X_{i,N}$ are attributes at day/month i which is described as follows:

$X_{i,1}$: BTCs in Circulation in month i is the total number of mined BTC currently circulating on the network.

$X_{i,2}$: Market capitalization in the month i is calculated by multiplying the total number of BTCs in circulation by the BTC price.

$X_{i,3}$*:* Block size in month i imposes a limit on the number of transactions that can be verified on each block. As a result of such a mechanism, larger blocks require more processing power and longer extraction time.

$X_{i,4}$: Average block size in month i.

$X_{i,5}$: Orphaned blocks in the month i are blocks that are not accepted into the blockchain network, which is created due to the delay in receiving a block, at which point another miner responds to the same block. Orphan blocks are valid, but do not register any transaction and have been rejected by the chain.

$X_{i,6}$: Number of transactions per block in month *i* are the transactions that happen in a block, and as soon as a block is solved, it is not possible to extend the block by adding more transactions.

$X_{i,7}$: Median confirmation time in month *i* is the median time for dealing with miners’ fees enclosed in a mined block and superimposed to the public ledger.

$X_{i,8}$: Hash rate in day/month *i* is the speed at which computational operations are completed to mine a BTC block.

$X_{i,9}$: Mining difficulty in month i is a measure of how difficult it is to mine a BTC block, or in more technical terms, to find a hash below a given target.

$X_{i,10}$: Transaction fees in month *i* are paid when cryptocurrencies are transferred to another wallet. Processing transactions on the blockchain takes effort, and these fees are used to compensate the miners and validators who help keep things running smoothly.

$X_{i,11}$: Cost per transaction in month *i* is calculated as miners’ revenue divided by the number of transactions.

$X_{i,12}$: Unique addresses in month *i* are installment addresses that have a non-zero adjust. This metric is one way of understanding day-by-day utilization of the BTC arrangement.

$X_{i,13}$: Total BTC transactions in month i.

$X_{i,14}$: Transaction volume excluding popular addresses in month i is the total number of transactions excluding those involving the network’s 100 most popular addresses.

$X_{i,15}$: Total output value in month i is the total value of all transaction outputs, including coins, returned to the sender as change.

$X_{i,16}$: Estimated transactions value in month i is the total estimated value in BTC transactions on the blockchain, which does not include coins returned as change.

$X_{i,17}$: Nasdaq Composite is a stock market index of the common stocks and similar securities listed on the Nasdaq stock market.

$X_{i,18}$: Dow Jones Industrial Average (DJIA) index in month i is a stock market index that measures the stock performance of 30 large companies listed on stock exchanges in the United States.

$X_{i,19}$: S&P500 stock market index in month i is a stock market index that measures the stock performance of 500 large companies listed on stock exchanges in the United States.

$X_{i,20}$: Gold-fixing price in month i is the setting of the gold price that takes place via a dedicated conference line. The price continues to be set twice daily at 10:30 and 15:00 London GMT in US dollars.

$X_{i,21}$: West Texas Intermediate crude oil (WTI) prices or Texas light sweet in month i is a benchmark in oil pricing, refined mainly in the Midwest and Gulf Coast regions in the United States.

$X_{i,22}$: US federal funds rate in month i is the interest rate at which depositors trade federal funds with each other at night. When a depository institution has a surplus in its reserve accounts, it can lend to other banks that need those funds. In other words, a bank with extra cash can lend it to another bank with a liquidity problem, and thus the cash balance of a bank with a problem with cash increases rapidly.

$X_{i,23}$: Breakeven inflation rate in month i is a measure of expected inflation, the difference between a nominal bond’s yield and an inflation-linked bond with the same maturity.

**Table A1:** Descriptive statistics.

Indicators	Min	Max	Mean	Std. Dev
BTCs in Circulation	4,002,626.667	17,213,768.33	12,440,527.97	3,742,297.323
Market Capitalization	280,390.572	2.55 × 10^11^	23,117,501,754	49,243,267,354
Block Size	1	179,101.0913	47,145.66415	54,242.32369
Average Block Size	0.01	1.054375	0.409168031	0.354286668
Orphaned Block	0	2.071428571	0.361061508	0.556278003
Transactions Per Block	1.625	2208.7575	760.5240094	666.8726595
Median Confirmation Time	6.201875	16.96133333	9.397754898	2.300005847
Hash Rate	0.01	49,050,545.4	3,657,003.427	9,072,465.431
Mining Difficulty	797.7186667	6.32 × 10^12^	4.79166 × 10^11^	1.18908 × 10^12^
Transaction Fee	0.056875	591.31625	62.77254092	105.0417027
Cost Per Transaction	1.242	117.1433333	20.31455332	25.94717866
Unique Addresses	513.6666667	825,390.9375	224,455.5763	207,905.1755
Total Transactions Per Day	464.0666667	358,831.0625	11,5921.8508	100,973.4369
Transaction Volume Excluding Popular Addresses	464.0666667	341,004.75	107,356.0276	101,413.1972
Total Output Value	63,281.56267	11,338,010.91	1,650,410.836	1,539,818.478
Estimated Transaction Value	27,539.66667	997,305.9375	209,259.0939	130,674.3663
Nasdaq Composite	2286.248	7882.400667	4435.937597	1480.271196
Dow Jones Industrial Average	10,576.508	25,807.52933	16,784.42003	3980.41153
S&P 500	1119.546667	2855.994	1880.952363	472.923476
Gold Price Index	1072.293333	1773.213333	1361.324703	184.7161172
Crude Oil WTI	30.485	110.3573333	74.41217956	23.3697773
US Federal Funds Rate	0.067142857	1.915333333	0.392972284	0.491999589
Breakeven Inflation Rate	1.302857143	2.586666667	2.011131634	0.298349738

**Table A2:** VIF for choosing attributes.

Variables	VIF
BTCs in Circulation	7.98
Market Capitalization	27.44 *
Block Size	7.68
Average Block Size	5.07
Orphaned Block	1.5
Transactions Per Block	39.11 *
Median Confirmation Time	1.73
Hash Rate	52.36 *
Mining Difficulty	51.45 *
Transaction Fee	3.53
Cost Per Transaction	33.88 *
Unique Addresses	9.75
Total Transactions Per Day	48.67 *
Transaction Volume Excluding Popular Addresses	8.44
Total Output Value	2.4
Estimated Transaction Value	2.49
Nasdaq Composite	11.86 *
Dow Jones Industrial Average	23.71 *
S&P 500	44.93 *
Gold Price Index	2.32
Crude Oil WTI	2.16
US Federal Funds Rate	1.99
Breakeven Inflation Rate	2.97

‘*’ VIF greater than 10.

**Table A3:** OLS regression results.

Variables	Model 1	Model 2	Model 3	Model 4	Model 5	Model 6	Model 7	Model 8	Model 9
BTCs in Circulation									
Block Size	0.268 ** (0.039)				0.521 * (0.164)	0.418 * (0.196)	0.408 ** (0.038)	0.443 ** (0.021)	0.436 ** (0.024)
Transaction Fees	0.131 *** (0.001)	0.167 ** (0.05)		0.158 ** (0.049)			0.166 *** (0.002)		0.155 ** (0.003)
Unique Addresses					1.021 *** (0.184)				
Total Number of Transactions			−0.023 . (0.012)						
Estimated Transaction Value	−0.096 ** (0.03)	−0.192 ** (0.071)		−0.179 * (0.070)	−0.149 * (0.062)	−0.242 ** (0.071)			−0.213 *** (0.004)
Cost Per Transaction	0.781 ** (0.07)								
Mining Difficulty		0.327 * (0.124)							
Market Capitalization			1.00 *** (0.002)						
Hash Rate				0.397 ** (0.126)					
Nasdaq Composite							0.809 . (0.1)		
Dow Jones Industrial Average								0.277 ** (0.024)	
S&P500									0.081 ** (0.038)
Adjusted ^R2^	0.91	0.67	0.81	0.73	0.68	0. 67	0.73	0.78	0.73
Residual Standard Error	0.044	0.1	0.001	0.023	0.087	0.10	0.099	0.089	0.089
*p*-value	<2.2 × 10^−16^	5.56 × 10^−11^	<2.2 × 10^−16^	<2.2 × 10^−16^	1.073 × 10^−14^	7.28 × 10^−10^	<2.2 × 10^−16^	<2.2 × 10^−16^	<2.2 × 10^−15^

‘***’ Significant at the 0.001 level, ‘**’ Significant at the 0.01 level, ‘*’ Significant at the 0.05 level, ‘.’ Significant at the 0.1 level.

### Appendix A.2. Model 2. Model Description

The purpose is to find a model that can approximate a target function by navigating the space of possible hypotheses (e.g., for ANN models, the space of hypotheses includes the network topology and hyperparameters) to predict the price changes for one day ahead. The target function can be written as:

(A2) $${\hat{\Delta p}}_{i+1}=f\left(\Delta X_{i1},\;\Delta X_{i2},\;\Delta X_{i3},\ldots ,\Delta X_{in}\right)$$

where ${\hat{\Delta p}}_{i+1}$ are the BTC price changes at day i+1. $\Delta X_{i1}\;to\;\Delta X_{in}$ are attributes at day i that might affect the price changes, which are described as follows:

$X_{i1}:$ Trade-weighted US dollar index or broad index (TWEXB) on day i is a measure of the value of the United States dollar relative to other world currencies.

$X_{i2}:$ Gold-fixing price on day i is the setting of the price of gold that takes place via a dedicated conference line. The price continues to be set twice daily at 10:30 and 15:00 London GMT in US dollars.

$X_{i3}:$ Dow Jones Industrial Average (DJIA) index on the day i is a stock market index that measures the stock performance of 30 large companies listed on stock exchanges in the United States.

$X_{i4}:$ Brent Crude oil price on day i is a primary trading classification of sweet light crude oil from the North Sea that is an important benchmark that defines the prices for oil worldwide.

$X_{i5}:$ West Texas Intermediate crude oil (WTI) prices or Texas light sweet, on day i is a benchmark in oil pricing, refined mainly in the Midwest and Gulf Coast regions in the United States.

$X_{i6}:$ Trades per minute on the day i is the number of BTCs traded in a minute.

$X_{i7}:$ Ask sum (5%) on day i, calculated as the amount of BTC on the order books waiting to be sold within a 5% range from the BTC price.

$X_{i8}:$ Bid sum (5%) on day i, calculated as the amount of USD on the order books waiting to buy BTC within a 5% range from the BTC price.

$X_{i9}:$ Bid–ask spread (10BTC) on day i is spread with 10 BTC slippage, i.e., with 10 BTC worth of orders removed from bids and from asks, which is calculated as $\frac{ask_{min}-bid_{max}}{ask_{min}}\times 100.$

$X_{i10}:$ Bid–ask spread (100BTC) on day i, i.e., with 10 BTC worth of orders removed from bids and from asks, which is calculated as

$$\frac{ask_{min}-bid_{max}}{ask_{min}}\times 100.$$

$X_{i11}:$ Buy0BTC on day i, defined as buy orders with an amount of less than 1 BTC.

$X_{i12}:$ Sell0BTC on day i, defined as sell orders with an amount of less than 1 BTC.

$X_{i13}:$ Buy1BTC on day i, defined as buy orders with an amount of 1 BTC.

$X_{i14}:$ Sell1BTC on day i, defined as sell orders with an amount of 1 BTC.

$X_{i15}:$ Buy5BTC on day i, defined as buy orders with an amount of 5 BTC.

$X_{i16}:$ Sell5BTC on day i, defined as sell orders with an amount of 5 BTC.

$X_{i17}:$ Buy10BTC on day i, defined as buy orders with an amount of 10 BTC.

$X_{i18}:$ Sell10BTC on day i, defined as sell orders with an amount of 10 BTC.

$X_{i19}:$ Momentum (MTM) (10 days) on day i is the difference between the price of BTC on day i and the BTC price on i−Nth day which is N = 10 in this model.

$X_{i20}:$ Commodity Channel Index (CCI), on day i, compares the price of BTC against its simple moving average and mean deviation of the price.

$X_{i21}:$ Volume on day i is the number of BTCs traded during a given period, which is one day in our model.

$X_{i22}:$ Simple moving average (SMA) on day i, calculated by adding recent prices and then dividing that by the number of periods, is five days for this model.

**Table A4:** Descriptive statistics.

Indicators	Min	Max	Mean	Std. Dev
TWEXB	91.45	96.87	93.90	1.25
Gold Fixing Price	1130.55	1304.55	1228.23	42.67
DJIA	17,888.28	21,271.97	20,055.42	951.69
Brent Crude Oil Price	41.61	56.34	51.27	3.57
WTI	43.29	54.48	50.13	2.84
Trades Per Minute	0.92	63.17	11.71	10.43
Ask Sum (5%)	750.97	6067.64	2737.32	1065.50
Bid Sum (5%)	567.64	5667.86	2378.06	989.71
Bid–Ask Spread (10BTC)	0.04	0.66	0.17	0.11
Bid–Ask Spread (100BTC)	0.30	2.90	0.78	0.45
Buy0BTC	767.00	41,552.00	8257.62	7121.62
Sell0BTC	559.00	49,411.00	8630.88	8091.82
Buy1BTC	160.00	7583.00	1820.29	1436.78
Sell1BTC	179.00	9272.00	1781.89	1600.48
Buy5BTC	35.00	2055.00	332.16	301.08
Sell5BTC	25.00	2553.00	354.77	365.67
Buy10BTC	1.00	685.00	94.53	86.32
Sell10BTC	2.00	838.00	93.58	109.68
Momentum	85.96	120.56	97.87	5.60
CCI	−351.04	524.63	87.53	111.17
Volume	1,538,729.58	134,500,681.52	22,138,504.67	22,622,391.54
SMA	631.17	2867.59	1150.82	508.79

**Table A5:** Chosen attributes by different feature selection techniques.

Attributes	VIF	Genetic Search	Evolutionary Search	Best First Search
TWEXB	✓			✓
Gold-Fixing Price	✓			
DJIA	✓			
Brent Crude Oil Price	✓	✓		✓
WTI				
Volume				
Trades Per Minute	✓			
Ask sum (5BTC)	✓			
Bid Sum (5BTC)				
Bid–Ask Spread (10BTC)	✓	✓	✓	✓
Bid–Ask Spread (100BTC)	✓	✓		✓
Buy0BTC				
Sell0BTC	✓			
Buy1BTC				
Sell1BTC	✓			
Buy5BTC				
Sell5BTC				
Buy10BTC	✓		✓	
Sum5BTCPrice		✓	✓	✓
Sell10BTC	✓		✓	
MTM	✓			
CCI	✓	✓	✓	✓

### Appendix A.3. Model 3. Model Description

The purpose is to find a model that can approximate a target function, which can be written as:

(A3) $${\hat{\Delta p}}_{i+1}=f\left(\Delta X_{i1},\;\Delta X_{i2},\;\Delta X_{i3},\ldots ,\Delta X_{in}\right)$$

where ${\hat{\Delta p}}_{i+1}$ are the BTC price changes at day i+1. $\Delta X_{i1}\;to\;\Delta X_{in}$ are attributes at day i that might affect the price changes, which are described as follows.

$X_{i1}:$ S&P500 stock market index on day i is a stock market index that measures the stock performance of 500 large companies listed on stock exchanges in the United States.

$X_{i2}:$ Dow Jones Industrial Average (DJIA) index on day i is a stock market index that measures the stock performance of 30 large companies listed on stock exchanges in the United States

$X_{i3}:$ CAC 40 stock market index on day i is a stock market index representing a capitalization-weighted measure of the 40 most significant stocks among the 100 most oversized market caps on the Euronext Paris.

$X_{i4}:$ West Texas Intermediate crude oil (WTI) prices or Texas light sweet on day i is a benchmark in oil pricing, refined mainly in the Midwest and Gulf Coast regions in the United States.

$X_{i5}:$ Nasdaq Composite on day i is a stock market index of the common stocks and similar securities listed on the Nasdaq stock market.

$X_{i6}:$ Gold-fixing price on day i is the setting of the gold price that takes place via a dedicated conference line. The price continues to be set twice daily at 10:30 and 15:00 London GMT in US dollars.

$X_{i7}:$ Bid–ask spread (10BTC), on day i is spread with 10 BTC slippage, that is with 10 BTC worth of orders removed from bids and from asks, which is calculated as $\frac{ask_{min}-bid_{max}}{ask_{min}}\times 100.$

$X_{i8}:$ Ask sum (10%) on day i, calculated as the amount of BTC on the order books waiting to be sold within a 10% range from the BTC price.

$X_{i9}:$ Bid sum (10%) on day *i*, calculated as the amount of USD on the order books waiting to buy BTC within a 10% range from the BTC price.

$X_{i10}:$ Trades per minute on day *i* are the number of BTCs traded in a minute.

$X_{i11}:$ Volatility on day i is the changes in market prices over a specified period. The faster prices change, the higher the volatility. It can be measured and calculated based on historical prices and can be used for trend identification.

$X_{i12}:$ Volume on day i is the number of BTCs traded during a given period, which is one day in our model.

$X_{i13}:$ Simple moving average (SMA) on day i, calculated by adding recent prices and then dividing that by the number of periods, which is five days for this model.

$X_{i14}:$ Hash rate on day i is the speed at which computational operations are completed to mine a BTC block.

$X_{i15}:$ Mining difficulty on day i is a measure of how difficult it is to mine a BTC block, or in more technical terms, to find a hash below a given target.

$X_{i16}:$ Number of transactions per block on day i are the transactions that happen in a block, and as soon as a block is solved, it is not possible to extend the block by adding in more transactions.

$X_{i17}:$ Block time on day i is an average time to mine a block in minutes.

**Table A6:** Descriptive statistics.

Data	Min	Max	Mean	Std. Dev
S&P500 Index	2581.00	2872.87	2711.50	63.73
Nasdaq Composite	6777.16	7637.86	7246.83	186.26
DJIA Index	23,533.20	26,616.71	24,842.04	676.33
CAC 40 Index	1425.12	5640.10	5297.19	527.43
WTI	59.19	72.24	65.03	3.34
Gold Fixing Price	1285.85	1360.25	1324.94	16.97
Bid/Ask Spread (10BTC)	0.21	0.68	0.36	0.12
Ask Sum (10%)	5.62 × 10^6^	2.32 × 10^7^	1.21 × 10^7^	3.62 × 10^6^
Bid Sum (10%)	9.71 × 10^6^	2.65 × 10^7^	1.49 × 10^7^	3.34 × 10^6^
Trades Per Minute	10.50	94.21	31.59	14.22
Volatility	7.80	154.64	39.35	25.15
Volume	3144.45	70961.37	3144.45	8830.17
SMA	1498.466429	14,907.4622	9030.497881	2036.55495
Hash Rate	1.63 × 10^18^	9.42 × 10^18^	3.89 × 10^18^	1.12 × 10^18^
Mining Difficulty	1.93 × 10^12^	4.94 × 10^12^	3.30 × 10^12^	7.27 × 10^11^
Number of Transactions Per Block	1.35 × 10^5^	4.25 × 10^5^	2.10 × 10^5^	5.16 × 10^4^
Block Time	7.48	12.22	9.34	0.86

**Table A7:** Attributes selected by different feature selection methods.

**Attributes**	**Best First Search**	**PSO** **Search**	**Evolutionary Search**
S&P500 Index			✓
Nasdaq Composite			
DJIA Index		✓	
CAC 40 Index	✓	✓	✓
WTI	✓		✓
Gold Fixing Price	✓		✓
Bid–Ask Spread (10BTC)	✓	✓	
Ask Sum within (10BTC)			✓
Bid Sum within (10BTC)			
Trades Per Minute	✓	✓	✓
Volatility	✓	✓	✓
Volume			
SMA	✓	✓	✓
Hash rate			
Mining Difficulty			
Number of Transactions			
Block Time	✓	✓	✓

## References

[1] Rosenberg J.M., Krist C. (2021). Combining machine learning and qualitative methods to elaborate students’ ideas about the generality of their model-based explanations. J. Sci. Educ. Technol..

[2] Bertolini R., Finch S.J., Nehm R.H. (2021). Testing the impact of novel assessment sources and machine learning methods on predictive outcome modeling in undergraduate biology. J. Sci. Educ. Technol..

[3] Ashayer A. (2019). Modeling and Prediction of Cryptocurrency Prices Using Machine Learning Techniques.

[4] Dutta A., Kumar S., Basu M. (2020). A gated recurrent unit approach to bitcoin price prediction. J. Risk Financ. Manag..

[5] Wang S., Vergne J.-P. (2017). Buzz factor or innovation potential: What explains cryptocurrencies’ returns?. PLoS ONE.

[6] Conrad C., Custovic A., Ghysels E. (2018). Long-and short-term cryptocurrency volatility components: A GARCH-MIDAS analysis. J. Risk Financ. Manag..

[7] Jang H., Lee J. (2017). An empirical study on modeling and prediction of bitcoin prices with bayesian neural networks based on blockchain information. Ieee Access.

[8] Chen Z., Li C., Sun W. (2020). Bitcoin price prediction using machine learning: An approach to sample dimension engineering. J. Comput. Appl. Math..

[9] Pang Y., Sundararaj G., Ren J. Cryptocurrency price prediction using time series and social sentiment data. Proceedings of the 6th IEEE/ACM International Conference on Big Data Computing, Applications and Technologies.

[10] Antoniou A., Ergul N., Holmes P., Priestley R. (1997). Technical analysis, trading volume and market efficiency: Evidence from an emerging market. Appl. Financ. Econ..

[11] Buchholz M., Delaney J., Warren J., Parker J. (2012). Bits and bets, information, price volatility, and demand for Bitcoin. Economics.

[12] Nai Fovino I., Steri G., Fontana A., Ciaian P., Kancs D., Nordvik J. (2015). On Virtual and Crypto Currencies: A General Overview, from the Technological Aspects to the Economic Implications.

[13] Ciaian P., Rajcaniova M., Kancs D.A. (2018). Virtual relationships: Short- and long-run evidence from BitCoin and altcoin markets. J. Int. Financ. Mark. Inst. Money.

[14] Keynes J.M. (1936). The General Theory of Employment, Interest, and Money.

[15] Kristoufek L. (2015). What are the main drivers of the Bitcoin price? Evidence from wavelet coherence analysis. PLoS ONE.

[16] Lyons R.K. (2001). The Microstructure Approach to Exchange Rates.

[17] Marshall A. (2013). Principles of Economics.

[18] Zhang Y.-C. (1999). Toward a theory of marginally efficient markets. Phys. A Stat. Mech. Its Appl..

[19] Amihud Y., Mendelson H. (1986). Asset pricing and the bid-ask spread. J. Financ. Econ..

[20] Reinganum M.R. (1990). Market microstructure and asset pricing: An empirical investigation of NYSE and NASDAQ securities. J. Financ. Econ..

[21] Dyhrberg A.H., Foley S., Svec J. (2018). How investible is Bitcoin? Analyzing the liquidity and transaction costs of Bitcoin markets. Econ. Lett..

[22] Scaillet O., Treccani A., Trevisan C. (2020). High-frequency jump analysis of the bitcoin market. J. Financ. Econom..

[23] Guo T., Bifet A., Antulov-Fantulin N. (2018). Bitcoin volatility forecasting with a glimpse into buy and sell orders. Proceedings of the 2018 IEEE International Conference on Data Mining (ICDM).

[24] Noble P.M., Gruca T.S. (1999). Industrial pricing: Theory and managerial practice. Mark. Sci..

[25] Hayes A.S. (2017). Cryptocurrency value formation: An empirical study leading to a cost of production model for valuing bitcoin. Telemat. Inform..

[26] Hayes A.S. (2019). Bitcoin price and its marginal cost of production: Support for a fundamental value. Appl. Econ. Lett..

[27] Yu Y., Rashidi M., Samali B., Yousefi A.M., Wang W. (2021). Multi-image-feature-based hierarchical concrete crack identification framework using optimized SVM multi-classifiers and D–S fusion algorithm for bridge structures. Remote Sens..

[28] Mohan S., Thirumalai C., Srivastava G. (2019). Effective heart disease prediction using hybrid machine learning techniques. IEEE Access.

[29] Li C., He Y., Xiao D., Luo Z., Fan J., Liu P.X. (2022). A novel hybrid approach of ABC with SCA for the parameter optimization of SVR in blind image quality assessment. Neural Comput. Appl..

[30] Ebrahimpour Z., Wan W., Khoojine A.S., Hou L. (2020). Twin hyper-ellipsoidal support vector machine for binary classification. IEEE Access.

[31] Yu Y., Li Y., Li J., Gu X. (2016). Self-adaptive step fruit fly algorithm optimized support vector regression model for dynamic response prediction of magnetorheological elastomer base isolator. Neurocomputing.

[32] Chun M.W., Huat N.C., Pauline O. (2022). Application of machine learning algorithm to prediction of thermal spring back of hot press forming. Res. Prog. Mech. Manuf. Eng..

[33] Shafiabady N., Lee L.H., Rajkumar R., Kallimani V., Akram N.A., Isa D. (2016). Using unsupervised clustering approach to train the Support Vector Machine for text classification. Neurocomputing.

[34] Erfanian S., Ziaullah M., Tahir M.A., Ma D. (2021). How does justice matter in developing supply chain trust and improving information sharing-an empirical study in Pakistan. Int. J. Manuf. Technol. Manag..

[35] Razzaq A., Tang Y., Qing P. (2021). Towards Sustainable Diets: Understanding the Cognitive Mechanism of Consumer Acceptance of Biofortified Foods and the Role of Nutrition Information. Int. J. Envion. Res. Pub. Health.

[36] Aggarwal D., Chandrasekaran S., Annamalai B. (2020). A complete empirical ensemble mode decomposition and support vector machine-based approach to predict Bitcoin prices. J. Behav. Exp. Financ..

[37] Jiang X. (2019). Bitcoin price prediction based on deep learning methods. J. Math. Financ..

[38] Munim Z.H., Shakil M.H., Alon I. (2019). Next-day bitcoin price forecast. J. Risk Financ. Manag..

[39] Huang J.-Z., Huang W., Ni J. (2019). Predicting bitcoin returns using high-dimensional technical indicators. J. Financ. Data Sci..

[40] Shen Z., Wan Q., Leatham D.J. (2019). Bitcoin Return Volatility Forecasting: A Comparative Study of GARCH Model and Machine Learning Model. J. Risk Financ. Manag..

[41] Mangla N., Bhat A., Avabratha G., Bhat N. (2019). Bitcoin price prediction using machine learning. Int. J.l Inf. Comput. Sci..

[42] Siami-Namini S., Namin A.S. (2018). Forecasting economics and financial time series: ARIMA vs. LSTM. arXiv.

[43] Pichl L., Kaizoji T. (2017). Volatility analysis of bitcoin. Quant. Financ. Econ..

[44] Indera N., Yassin I., Zabidi A., Rizman Z. (2017). Non-linear autoregressive with exogeneous input (NARX) Bitcoin price prediction model using PSO-optimized parameters and moving average technical indicators. J. Fundam. Appl. Sci..

[45] Razzaq A., Liu H., Zhou Y., Xiao M., Qing P. (2022). The Competitiveness, Bargaining Power, and Contract Choice in Agricultural Water Markets in Pakistan: Implications for Price Discrimination and Environmental Sustainability. Front. Environ. Sci..

[46] Rosenblatt F. (1958). Two Theorems of Statistical Separability in the Perceptron.

[47] Smola A.J., Schölkopf B. (2004). A tutorial on support vector regression. Stat. Comput..

[48] Vapnik V. (1999). The Nature of Statistical Learning Theory.

[49] Hansen L.K., Salamon P. (1990). Neural network ensembles. IEEE Trans. Pattern Anal. Mach. Intell..

[50] Wright S. (1921). Correlation and Causation. J. Agric. Resour..

[51] Hyndman R.J., Koehler A.B. (2006). Another look at measures of forecast accuracy. Int. J. Forecast..

[52] Kuhn M., Johnson K. (2013). Applied Predictive Modeling.

